# Electrospray Ionization Efficiency Predictions and Analytical Standard Free Quantification for SFC/ESI/HRMS

**DOI:** 10.1021/jasms.3c00156

**Published:** 2023-06-26

**Authors:** Stefan Bieber, Thomas Letzel, Anneli Kruve

**Affiliations:** † AFIN-TS GmbH (Analytisches Forschungsinstitut für Non-Target Screening), Am Mittleren Moos 48, 86167 Augsburg, Germany; ‡ Department of Materials and Environmental Chemistry, Stockholm University, Svante Arrhenius Väg 16, 10691 Stockholm, Sweden; § Department of Environmental Science, Stockholm University, Svante Arrhenius Väg 16, 10691 Stockholm, Sweden

**Keywords:** supercritical fluid chromatography, nontarget screening, quantification, machine learning, ionization efficiency

## Abstract

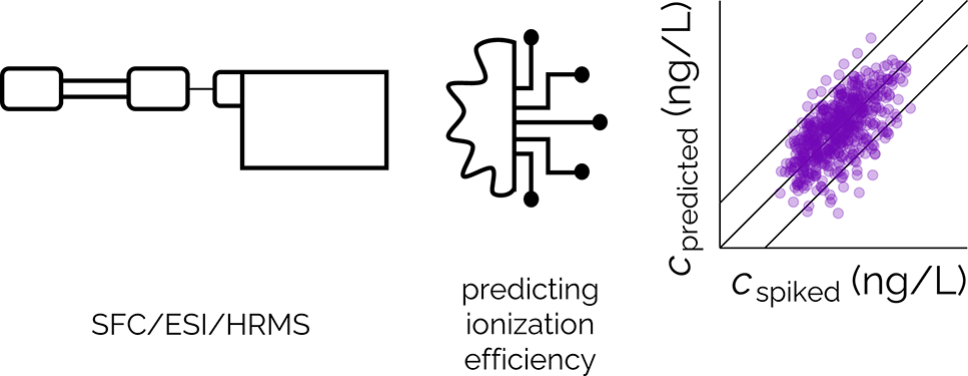

Supercritical fluid chromatography (SFC) is a promising, sustainable, and complementary alternative to liquid chromatography (LC) and has often been coupled with high resolution mass spectrometry (HRMS) for nontarget screening (NTS). Recent developments in predicting the ionization efficiency for LC/ESI/HRMS have enabled quantification of chemicals detected in NTS even if the analytical standards of the detected and tentatively identified chemicals are unavailable. This poses the question of whether analytical standard free quantification can also be applied in SFC/ES/HRMS. We evaluate both the possibility to transfer an ionization efficiency predictions model, previously trained on LC/ESI/HRMS data, to SFC/ESI/HRMS as well as training a new predictive model on SFC/ESI/HRMS data for 127 chemicals. The response factors of these chemicals ranged over 4 orders of magnitude in spite of a postcolumn makeup flow, expectedly enhancing the ionization of the analytes. The ionization efficiency values were predicted based on a random forest regression model from PaDEL descriptors and predicted values showed statistically significant correlation with the measured response factors (*p* < 0.05) with Spearman’s rho of 0.584 and 0.669 for SFC and LC data, respectively. Moreover, the most significant descriptors showed similarities independent of the chromatography used for collecting the training data. We also investigated the possibility to quantify the detected chemicals based on predicted ionization efficiency values. The model trained on SFC data showed very high prediction accuracy with median prediction error of 2.20×, while the model pretrained on LC/ESI/HRMS data yielded median prediction error of 5.11×. This is expected, as the training and test data for SFC/ESI/HRMS have been collected on the same instrument with the same chromatography. Still, the correlation observed between response factors measured with SFC/ESI/HRMS and predicted with a model trained on LC data hints that more abundant LC/ESI/HRMS data prove useful in understanding and predicting the ionization behavior in SFC/ESI/HRMS.

## 1 Introduction

Suspect and nontarget screening (NTS) with liquid chromatography electrospray high resolution mass spectrometry (LC/ESI/HRMS) is increasingly used in environmental screening,^1^ human biomonitoring,^2^ metabolic phenotyping,^3^ medical devices,^4^ as well as process monitoring.^5^ The results from sample analysis without the authentic calibration standards are intrinsically qualitative due to the fact that the ionization efficiency of chemicals in an ESI source may range over 6 or 7 orders of magnitude.^6^ The ionization efficiency is affected by the properties of the analyte^6,7^ as well as the mobile phase.^8^ Generally, more polar analytes possess lower ionization efficiency while chemicals with larger nonpolar moiety possess higher ionization efficiency,^6,9−11^ as long as sufficient basicity/acidity is available for protonation/deprotonation.^12^ Simultaneously, higher organic modifier content in the mobile phase improves the ionization efficiency^8,13^ due to faster evaporation of the mobile phase and drying of ESI droplets, therefore reaching faster the Coulomb explosion and producing faster more ions (in case the molecule weights are below 1000 Da).^14^ Additionally, the pH^13^ and buffer type^15,16^ of the mobile phase strongly affect the ionization efficiency.

Recently, predicting the ionization efficiency of chemicals with machine learning has enabled turning peak areas into concentration estimates with so-called semiquantification.^17−20^ Quantification based on predicted ionization efficiency has been already used in NTS employing reversed phase LC for analysis of emerging contaminants,^21^ including transformation products,^18^ in surface water, pesticide residues in food,^17^ as well as persistent pollutants in exposure studies.^22^ These ionization efficiency prediction models account for the mobile phase composition (pH, buffer type, organic modifier and its contents) at the time of elution^17^ and are therefore expected to be independent of the chromatographic separation. Still, research and application have so far been exclusively carried out for reversed phase liquid chromatography.

Supercritical fluid chromatography (SFC) is an environmentally more sustainable alternative to LC, where carbon dioxide, with or without an organic modifier, is used to carry the analytes through packed columns. Due to the unique characteristics of its mobile phase, SFC provides fast and efficient separations.^23^ The polarity range of separable chemicals in SFC can be significantly broader than in individual LC techniques, e.g., reversed phase (RP) or hydrophilic interaction chromatography.^24^ As a result, it is applicable for the nontargeted comprehensive analysis of complex samples. Coupling SFC with HRMS for NTS has enabled the detection of various contaminants of emerging concern in surface waters by Bieber et al.,^24^ 20 novel micropollutants in groundwater by Tisler et al.,^25^ as well as analysis of oxygenated polycyclic aromatic chemicals in oils^26^ and monitoring of the quality of chocolate.^27^ In spite of the obvious similarities of RPLC/ESI/HRMS and SFC/ESI/HRMS, semiquantification has only been applied to the former.

In order to assess the applicability of semiquantification in SFC/ESI/HRMS, we investigated the response factor of 127 chemicals as well as evaluated the possibility to predict the response factor. We investigated two approaches. First, we predicted response factors based on existing models trained for LC/ESI/HRMS.^17,28^ Second, we trained and validated a random forest regression model directly on the SFC/ESI/HRMS data. We evaluated the possibility of quantifying small molecules detected with SFC/ESI/HRMS based on the ionization efficiency values predicted with both models. To shed light on the properties of the chemicals that affect the ionization efficiency in both SFC/ESI/HRMS and LC/ESI/HRMS, we furthermore compared the significant descriptors in both models. Finally, we investigated the possibility to transfer previously trained LC/ESI/HRMS models to SFC/ESI/HRMS by Monte Carlo sampling of calibration chemicals and evaluated the importance of covering a wide range of ionization efficiency values and retention time.

## 2 Materials and Methods

### 2.1 SFC/ESI/HRMS Method

The data was generated by SFC (Agilent Technologies, Waldbronn, Germany) with a method using carbon dioxide (A) vs methanol with 20 mmol/L ammonium acetate (B). The mobile phase composition was kept constant at 2% of B for 1 min and then increased to 60% within 6 min, kept at 60% for 4 min, and then lowered back to 2% within 0.1 min. The postrun time was 1.9 min. The mobile phase flow rate was 1.500 mL/min. Before entering the ESI source, a constant makeup flow of 200 μL/min water/isopropanol 90%/10% was added to the SFC eluting flow.

The stationary phase was a zwitterionic Eurosphere II HILIC column with 150 × 3 mm, 3 μm particles (fully porous) (KNAUER, Berlin, Germany). The mass spectrometric data was acquired with Exploris 120 high-resolution Orbitrap mass spectrometer (Thermo Fisher Scientific GmbH; Dreieich, Germany) in full scan mode from 67 to 1000 *m*/*z* at a resolution of 60,000. Sheath gas, aux gas, and sweep gas flow rates were set at 50, 8 and zero arb. units. The vaporizer temperature was set to 350 °C, and the ion transfer tube was heated to 320 °C. Capillary voltages were 3500 V in positive ionization. For data processing, TraceFinder software version 5.1 (Thermo Fisher Scientific GmbH; Dreieich, Germany) was utilized.

### 2.2 Analytes and Calibration Graphs

A standard mixture with a concentration of 1000 μmol/L in acetonitrile for chemicals with positive log *D* (pH 7) values and acetonitrile/water 50/50 (v/v) for chemicals with negative log *D* (pH 7) values. The concentration levels of the calibration solutions ranged between 12.2 to 1750 nmol/L in acetonitrile/water 50/50 (v/v). All solutions were analyzed in triplicate. The peaks were integrated for 137 chemicals in electrospray positive ionization mode with TraceFinder software.

The retention time agreed within 0.2 min and peak area within 30% for all of the detected chemicals. All peak areas were corrected for the natural isotope abundance to account for the fact that, in TraceFinder, only the main peak was integrated. For all detected chemicals, the linear range was evaluated manually based on the residuals of the calibration graph. In the linear range, the slope of the calibration graph of isotope corrected signal vs molar concentration was calculated and is denoted as the response factor (RF). Finally, 127 analytes (log *P* −4.5 to 5.9) yielded at least three concentrations in the linear range, and the RF values for these chemicals ranged from 4.06 × 10^14^ to 2.70 × 10^18^ M^–1^.

### 2.3 Chemical and Mobile Phase Descriptors

For all analytes, the PaDEL descriptors^29^ were computed with an in-house modified script, previously described by Kruve et al.,^18^ yielding in total 1217 descriptors. Among these descriptors are log *P* descriptors, polarity descriptors, and descriptors related to aromaticity, ring count, atom count, bind count, carbon type, electrotopological states, hydrogen bonding, etc. For predicting the ionization efficiency with a previously trained model suggested by Liigand et al.^17^ and retrained by Kruve et al.,^28^ 237 PaDEL descriptors were used. These descriptors have been selected as part of training the random forest regression by Kruve et al.^28^ For training a SFC specific prediction model, the descriptors with near zero variance (95/5 or larger ratio of most common to next most common value) were removed. Additionally, of the descriptors with a pairwise correlation coefficient higher than 0.75, only one descriptor with the lower mean absolute correlation of the remaining variable was kept in the data set.

Additionally, the ionization efficiency is known to depend on the mobile phase composition. Therefore, viscosity,^30^ surface tension,^31^ and polarity index^32^ were computed based on the mobile phase composition at the time of elution for each chemical and added to the data set alongside the pH and presence/absence of NH_4_+ ions in the mobile phase. Since it is very challenging to determine the pH of the water–organic mixtures and the pH is dependent on the fraction of organic modifier in the mobile phase, we refer here and below to the pH of the water phase.

### 2.4 Predicting Ionization Efficiency with a Pretrained Model

The ionization efficiency was predicted from the PaDEL descriptors and mobile phase descriptors with a random forest model previously published by Liigand et al.^17^ and for which the scope has been further widened by Kruve et al.^28^ This model has been trained on data collected with ESI/HRMS with flow injection analysis as well as reversed phase LC for 775 unique chemicals. Many of these chemicals, however, have been measured under various mobile phase conditions to enable independence of the ionization efficiency predictions from the chromatographic conditions used for specific analysis.

The predicted ionization efficiency values are relative (in positive ESI mode, benzoic acid is assigned as log IE = 0) and not instrumentation specific; therefore, the response factors of the 127 chemicals were used to transform the predicted ionization efficiency values to instrumentation specific response factors. The experimental RF values were correlated with the predicted ionization efficiency values log IE_pred_ with robust linear regression (*rlm* function from MASS package in R):
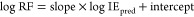
1and the slope and intercept were used to convert the predicted ionization efficiency values into instrument-specific response factors.

### 2.5 Modeling Response Factor for SFC

In addition to the evaluation of the previously trained ionization efficiency model, we also trained a model based on the RF values of the 127 chemicals measured in this study. As the data set is limited and using 20% of the data for validation would strongly bias the evaluation due to the random selection of the chemicals a 10-fold cross-validation was used instead. The data was divided into ten subsets so that each chemical was present in only one of the subsets. Additionally, the distribution of the data in each of the folds should represent the whole distribution as closely as possible. Therefore, the splitting was based on the ranked RF values. Nine of the subsets were used to train a random forest regression model (*RRF* function) with the *caret* package in R. The one remaining subset was used for validation of the model performance by predicting the ionization efficiency for this subset. The training was repeated ten times, using each subset once for validation, allowing to validate the performance on all 127 chemicals.

Random forest regression (*RRF*) was used for unambiguous comparison with the *RRF* model pretrained on LC/ESI/HRMS data. Additionally, the hyperparameters (*mtry*, *coefReg*, *coefImp*) of the random forest regression were optimized as part of the model training with the *train* function in the *caret* package in R. A two-by-two repeated cross-validation was used in the hyperparameter training. The optimal values were *mtry* = 86, *coefReg* = 1.0, and *coefImp* = 0.5. In case of SFC/ESI/HRMS model the response factors were predicted directly and no transfer of the values was required.

### 2.6 Concentration Prediction

With both models, the concentrations were predicted as follows:
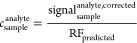
2To evaluate the accuracy of the predicted concentrations, an error factor was used as follows:
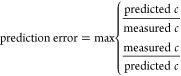
3The error factor enables comparison of prediction accuracies at low, medium, and high concentration levels as well as to equal treatment of over- and underestimation of fold prediction errors. The data are available in the Supporting Information.

## 3 Results and Discussion

### 3.1 Response Factor Trends

**Figure 1:**
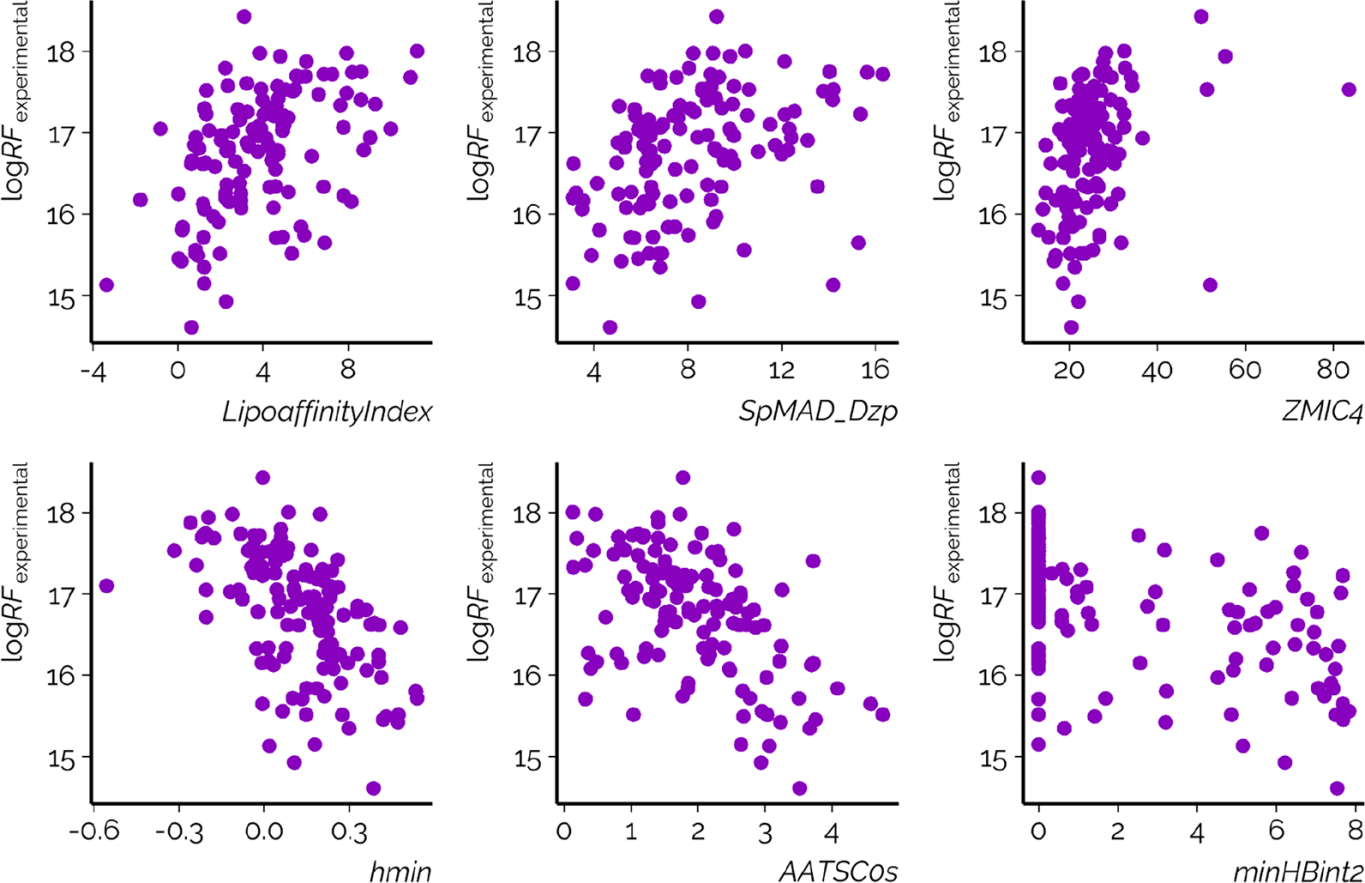
Strongest positive correlation with logarithmic RF values was observed for *LipoaffinityIndex*, *SpMAD_Dzp*, and *ZMIC4*, while the strongest negative correlation was found for *hmin*, *AATSC0s*, and *minHBint2*.

The impact of the properties of the chemicals and the mobile phase on the response factor in SFC/ESI/HRMS was studied based on the Spearman’s rho of the individual PaDEL descriptors and logarithmic measured RF values. The highest positive correlation was observed for *LipoaffinityIndex*, *SpMAD_Dzp*, and *ZMIC4* (Figure 1), where both *LipoaffinityIndex* and *SpMad_Dzp* are associated with the polarity of the molecule. The correlation of the measured RF values was strongest with *LipoaffinityIndex* yielding a Spearman’s rho of 0.49. This coincides with the previous findings from flow injection analysis and reversed phase LC/ESI/HRMS analysis that molecules with larger hydrophobic areas tend to possess higher response factors in ESI/MS.^6,10,11^ Chemicals with larger hydrophobic moieties also have higher *LipoaffinityIndex* and partition to the surface of the electrospray droplets, which facilitates the partitioning to the gas phase and yields higher ionization efficiency.^7,10^ Therefore, although the chromatographic separation mechanism in SFC differs significantly from that in RPLC, the patterns in ESI/MS response factors follow similar trends. This also encourages the investigation of the previously developed ionization efficiency prediction model for application in SFC/ESI/MS.

The strongest negative correlation was observed with *hmin*, *AATSC0s*, and *minHBint2* with Spearman’s rho values of −0.55, −0.49, and −0.48, respectively. Both *hmin* and *minHBint2* are Electrotopological State Atom Type descriptors and can be associated with the hydrogen bonding of the chemical. Previously, we have found that chemicals with higher hydrogen bond basicity show lower ionization efficiency in the electrospray negative mode. This can be explained by the fact that chemicals yielding more hydrogen bonds are less likely to partition to the surface of the ESI droplets and, therefore, form gas phase ions. Therefore, although the chromatographic separation mechanism in SFC differs significantly from that in reversed phase LC, the patterns in ESI/HRMS response factors follow similar trends. This also encourages investigation of the previously developed ionization efficiency prediction model for application in SFC/ESI/HRMS.

Furthermore, the increase in organic modifier content at the time of elution is known to further increases the ionization efficiency in LC/ESI/HRMS due to increased drying rate of the ESI droplets, leading to a response factor dependence on the retention time.^8,9,13^ In the current SFC/ESI/HRMS data set, Table S1, a weak negative correlation between the experimental RF values and retention time was observed with Spearman’s rho of −0.28.

### 3.2 Predicting Ionization Efficiency

**Figure 2:**
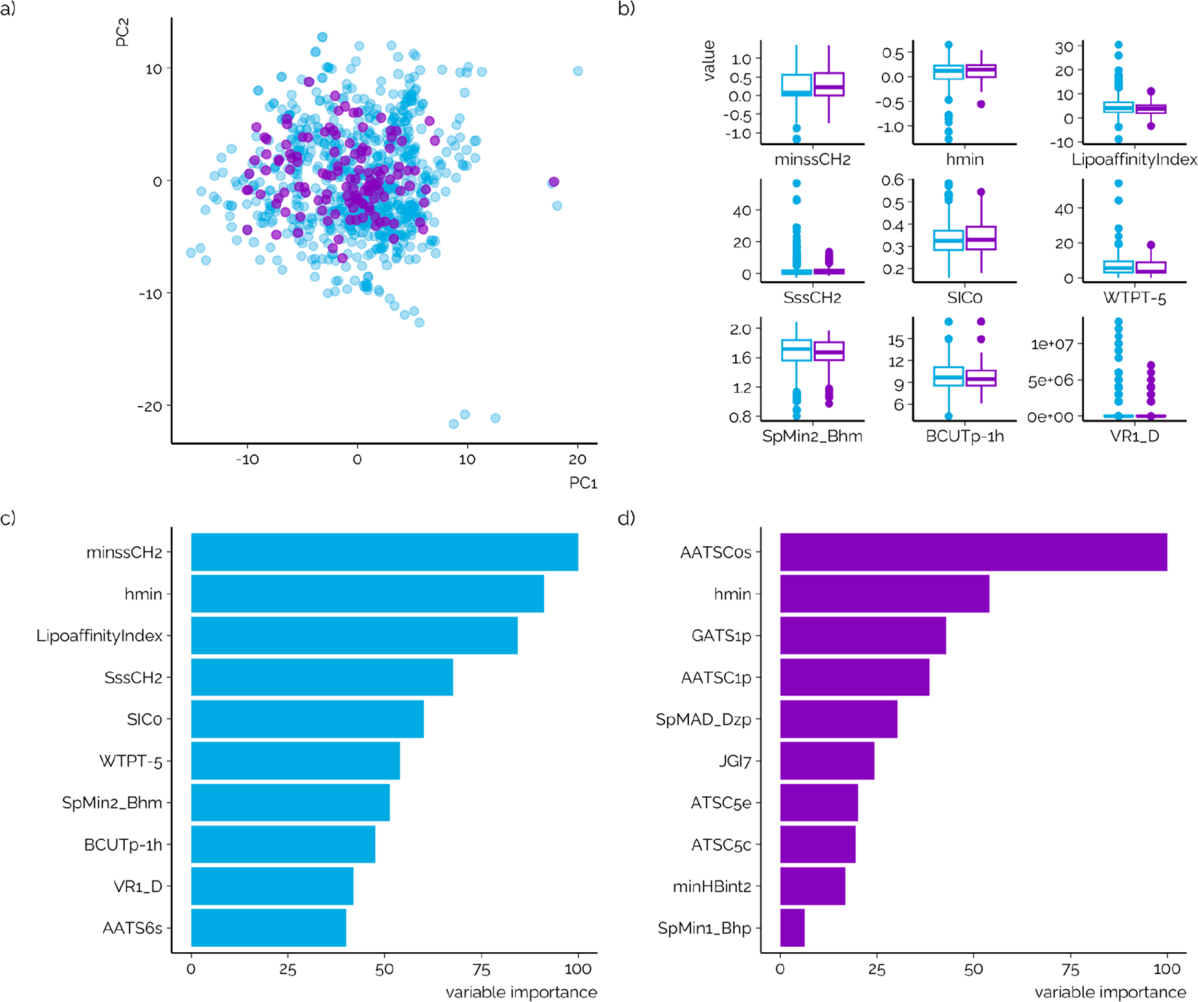
(a) Overlap in the chemical space of the data used previously for training the LC/ESI/HRMS model (blue dots) and used in this study (purple dots) based on the first two principal components (explaining ∼20% of total variance). (b) Overlap in individual descriptors with highest importance in the pretrained ionization efficiency prediction model.^17,28^ (c, d) Ten most influential descriptors for predicting ionization efficiency for LC/ESI/HRMS data (c, blue bars)^17,28^ and SFC/ESI/HRMS (d, purple bars).

First, principal component analysis (PCA) was used to evaluate the overlap in chemical space of the pretrained mode and chemicals considered here. All PaDEL descriptors included in the pretrained model were used in PCA. Based on the overlap in data points (Figure 2a) the pretrained model covers a wider chemical space. Furthermore, the comparison of individual descriptor values for the ten most influential descriptors (Figure 2b) also indicated a good overlap.

The ionization efficiencies were predicted for all chemicals with a random forest model previously developed by Liigand et al.^17^ and updated by Kruve et al.^18^ The predicted ionization efficiency values ranged from 1.30 to 4.20 logarithmic units. In comparison, the ionization efficiency values used for training the predictive model by Liigand et al.^17^ ranged from −1.0 to 6.5 logarithmic units. A narrower range is expected here due to the slightly narrower coverage of the chemical space (Figure 2a). The low ionization efficiency chemicals in the model training by Liigand et al.^17^ are primarily weak oxygen bases that are neutral even in acidic mobile phase. On the other hand, the chemicals with the highest ionization efficiencies were superbases with large hydrophobic moieties. Both of the edges are missing from the current data set mainly focusing on environmental contaminants.

**Figure 3:**
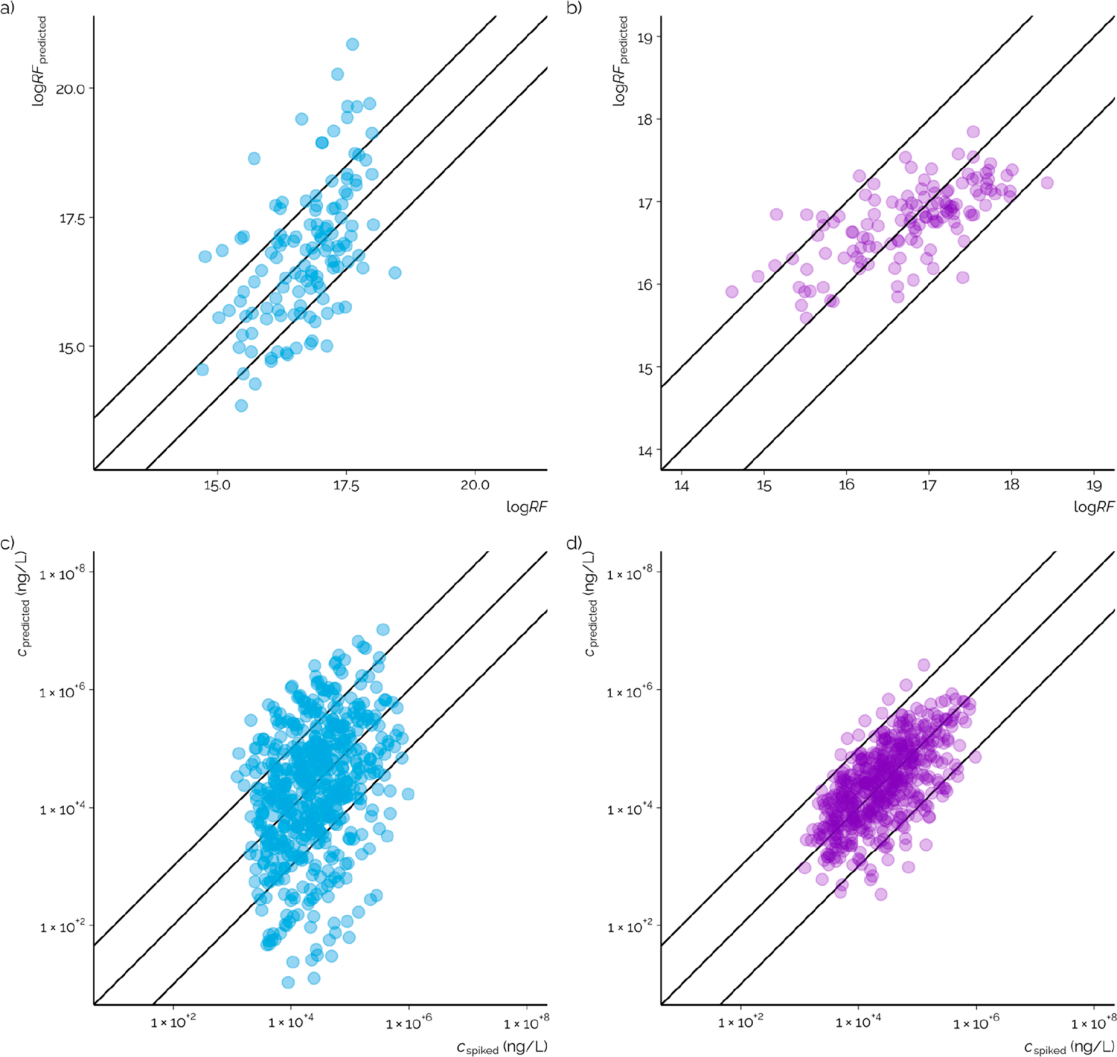
(a) Correlation of log IE values predicted with the random forest regression model pretrained on LC/ESI/HRMS data with logarithmic RF values from SFC/ESI/HRMS measurements. The lines show ideal agreement and ±1 order of magnitude confidence line. (b) Correlation of log IE values predicted with the random forest regression model trained on SFC/ESI/HRMS data with logarithmic RF values from SFC/ESI/HRMS measurements. (c, d) Correlation of concentration estimated from the predicted response factors vs the spiked concentration for both pretrained model and the model trained here, respectively. The lines show ideal agreement and ±1 order of magnitude confidence line.

The predicted ionization efficiency values were converted to predicted response factors by a robust linear regression (eq 1) between the predicted ionization efficiency values and experimental RF values. The experimental RF values (4.06 × 10^14^–2.70 × 10^18^ M^–1^) and predicted values (7.08 × 10^13^–7.08 × 10^20^ M^–1^) showed a statistically significant correlation with *p* value <2.1 × 10^–16^ and Spearman’s rho of 0.58 (Figure 3a). The outliers were amitriptyline and diphenhydramine for which two of the highest predicted log IE values were assigned, and the response factors were by a factor of 1000× overestimated. These structures show significant structural similarities with large aromatic moieties and a tertiary amine group, both have been observed to contribute to high ionization efficiency values in LC/ESI/HRMS.^6^

The observed correlation of predicted and experimental RF values indicates that a model previously trained on LC/ESI/HRMS data has the potential for application on a new chromatographic system. This can be associated with the fact that a large portion of the ionization efficiency measurements used for training the models^17^ have been collected with flow injection analysis and a full factorial design has been implemented for studying the mobile phase impact. Such a design allows the model to learn the impact of the chemical properties of the analyte and mobile phase properties independently. As a result, the application is independent of the used chromatographic mobile phases as long as the ionization mechanism is similar.

Furthermore, we were interested in evaluating how much the prediction accuracy of RF values could be improved by training the models directly on data acquired by SFC/ESI/HRMS. Due to a lower number of data (127 chemicals vs 775 chemicals), a 10-fold cross-validation was used to allow comparing the prediction accuracy for the same chemicals. The predicted response factors ranged from 3.92 × 10^15^ to 7.04 × 10^17^ M^–1^ (Figure 3b). The maximum difference in experimental and predicted response factors was a factor of 5, observed for methylisothiazolinone, while no apparent reasons could be pinpointed. All in all, the model trained on SFC/ESI/HRMS data showed considerably improved prediction accuracy compared to that of the model trained on LC/ESI/HRMS data. This probably arises from two reasons. First, the mobile phase pressure entering the ionization source is different for ESI/HRMS coupled with LC and SFC. Namely, the pressure of the mobile phase entering ESI source for SFC is 100–130 bar while for LC it is atmospheric pressure. This can cause mechanistic differences in the ionization process. Second, the model transfer to new instruments always introduces some additional error as observed also previously for different flow injection ESI/HRMS analysis while applying the same mobile phase composition.^33^ This is likely to stem from slight differences in the ESI source geometry, gas flows, applied voltages, etc. which somewhat affect the ionization process.

Several similar or overlapping descriptors were found to be important in the random forest model previously trained on LC/ESI/HRMS data and the model trained on SFC/ESI/HRMS data here. Namely, electroptopological state atom type descriptor smallest hydrogen E-State value (*hmin*) was top ranked for both models (Figure 2c, d). Also, Burden modified eigenvalue descriptors (*SpMin2_Bhm* and *SpMin1_Bhp*) were among top important descriptors and have been associated with the intermolecular interactions.^34^ In addition, several autocorrelation descriptors weighted over different charge descriptors (*AATSC0s*, *AATSC 1p*, *ATSC5e*, and *ATSC5c*) and therefore can be associated with the polarity of the chemicals. In the model pretrained on LC/ESI/HRMS data *LipoaffinityIndex* has a high rank and contributes with similar information to the model predictions. Therefore, the descriptors with high variable importance in both models share significant similarities and also agree with the empirical evidence found previously about the electrospray ionization efficiency dependence on the structure of the chemical.

### 3.3 Predicting Concentration

Both the pretrained model and the model trained on SFC/ESI/HRMS data were used to predict the concentrations of all of the 127 chemicals studied here (Figure 3c and d). The median concentration prediction error for the pretrained model was 5.11× and for the model trained here 2.20×. These performance characteristics agree well with the performance observed recently for reversed phase LC/ESI/HRMS, where mean prediction errors of 1.7×^21^ to 5.1×^35^ have been observed, depending on the complexity of the matrix. Similar to the predicted response factors, the model trained on the SFC/ESI/HRMS data showed a significantly improved accuracy. The mean prediction errors were 49.5× and 4.10×, respectively. In the case of pretrained model, the large mean error primarily stems from a large prediction error for a few chemicals. For example, the concentration prediction error for amitriptyline and diphenhydramine on all studied concentrations was 780× to 1900×. The response factors for these chemicals were considerably overestimated, and concentration therefore underestimated. However, the prediction errors generally are higher for the pretrained model, where 63% and 88% of the chemicals had a prediction error under 10× at all concentration levels for the pretrained model and model trained here, respectively. All in all, the model trained on SFC/ESI/HRMS data demonstrates prediction accuracy promising for application in quantification for NTS.

### 3.4 Choosing the Calibration Chemicals

**Figure 4:**
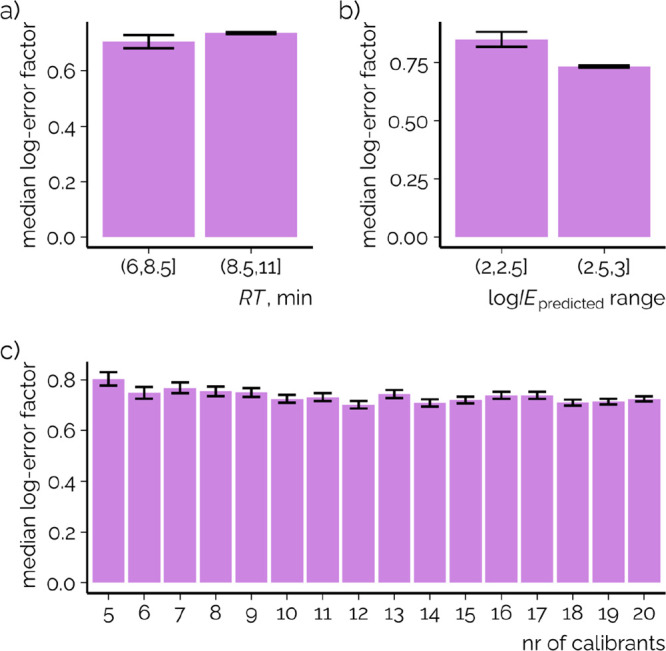
Dependence of the median concentration prediction error on the range of calibration chemicals’ (a) retention time, (b) predicted log IE range, and (c) number of chemicals used observed with the Monte Carlo simulation for sampling the chemicals for transferring the predicted ionization efficiency values to RF values. Monte Carlo simulations were repeated 200 times; bars show the mean of the median prediction error for each simulation, and error bars show one standard deviation of the mean of the median prediction error.

Due to the fact that both the model trained on LC/ESI/HRMS data and the model trained on SFC/ESI/HRMS data showed significant similarities in descriptor importance, it is of interest how much effect the model transfer process can have on the quantification accuracy. Therefore, we systematically evaluated the impact of a selection of calibration chemicals with Monte Carlo sampling. A 200-fold Monte Carlo sampling of five to 20 chemicals for the calibration was used, while the rest of the chemicals (122 to 107 chemicals, test set) were left for the evaluation of quantification prediction error. Furthermore, we evaluated the impact of the retention time range and predicted ionization efficiency range of these chemicals on the quantification error based on the median error factor for the test set chemicals. Interestingly, wide coverage of retention times was found to be unnecessary in choosing the calibration chemicals (Figure 4a). The low impact on the average prediction accuracy might be associated with the fact that in the experiments a makeup flow was added postcolumn, which possibly reduces the effect of the changing mobile phase composition. Additionally, log *P* showed only a weak relationship with the retention time, and therefore, it seems that in the case of SFC even close eluting calibration chemicals might provide sufficiently wide chemical diversity and are suitable for converting log IE_pred_ values to response factors.

It was observed that a larger range of predicted ionization efficiency values of calibration chemicals yielded a lower prediction error on average (Figure 4b) and that on average the prediction errors were very similar (Figure 4a) for 5–20 calibration chemicals with a slight decrease in prediction error with increasing number of chemicals. Even more importantly, the prediction error became more stable between the different random samples with the increasing number of chemicals used. While using only five chemicals, the standard deviation between samples was 0.372 log-units while for 20 chemicals it was 0.147 log-units. This is expected as in the case of a low number of chemicals each chemical has more weight in the linear regression (eq 2) used to transfer the predicted ionization efficiency values to RF values. The same was observed for the range of the predicted ionization efficiency values: a wider range yielded more stable prediction errors.

## 4 Conclusions

Here we have investigated the applicability of machine learning models trained on flow injection and reversed phase LC/ESI/HRMS data to predict ionization efficiency for SFC/ESI/HRMS. We observed a good correlation, indicating that a smart study design in data collection can enable training chromatography independent models, which can be further explored for other chromatography types and column chemistries. However, the performance was considerably improved by retraining the model on the SFC/ESI/HRMS data collected on the same instrument. Furthermore, the predicted ionization efficiency values could be used to predict the concentration with a median prediction error of 2.20× and hold great promise for quantification in NTS SFC/ESI/HRMS where analytical standards of detected and identified chemicals are scarcely available.

## Supporting Information Available

Analytes, respective retention times, isotope corrected signals, and respective concentrations as well as predicted concentrations (XLSX)
